# Mastering the scales: a survey on the benefits of multiscale computing software

**DOI:** 10.1098/rsta.2018.0147

**Published:** 2019-02-18

**Authors:** Derek Groen, Jaroslaw Knap, Philipp Neumann, Diana Suleimenova, Lourens Veen, Kenneth Leiter

**Affiliations:** 1 Department of Computer Science, Brunel University London, Uxbridge, UK; 2 US Army Research Laboratory, Aberdeen Proving Ground, Aberdeen, MD, USA; 3 Department of Scientific Computing, University of Hamburg, Hamburg, Germany; 4 Netherlands eScience Center, Amsterdam, The Netherlands

**Keywords:** multiscale computing, multiscale modelling, multiscale simulation, high-performance computing, usability

## Abstract

In the last few decades, multiscale modelling has emerged as one of the dominant modelling paradigms in many areas of science and engineering. Its rise to dominance is primarily driven by advancements in computing power and the need to model systems of increasing complexity. The multiscale modelling paradigm is now accompanied by a vibrant ecosystem of multiscale computing software (MCS) which promises to address many challenges in the development of multiscale applications. In this paper, we define the common steps in the multiscale application development process and investigate to what degree a set of 21 representative MCS tools enhance each development step. We observe several gaps in the features provided by MCS tools, especially for application deployment and the preparation and management of production runs. In addition, we find that many MCS tools are tailored to a particular multiscale computing pattern, even though they are otherwise application agnostic. We conclude that the gaps we identify are characteristic of a field that is still maturing and features that enhance the deployment and production steps of multiscale application development are desirable for the long-term success of MCS in its application fields.

This article is part of the theme issue ‘Multiscale modelling, simulation and computing: from the desktop to the exascale’.

## 1. Introduction

Many phenomena in science and engineering are amenable to multiscale modelling. Multiscale modelling is a divide-and-conquer paradigm in which multiscale models are built as assemblies of individual unit processes, often also referred to as at-scale models, operating at distinct spatial or temporal scales. With the inclusion of relevant unit processes, multiscale models are capable of accurately characterizing phenomena in regimes not easily observed *in vivo* or *in vitro*. Multiscale modelling is primarily a computational endeavour and, over the last two decades, a range of supporting software has emerged for building computational multiscale models, for example, facilitating the *coupling* of existing at-scale models, enabling the use of (remote) high-performance computing resources, or simplifying the management of multiscale simulation runs through automation. Although current *multiscale computing software* (MCS) has been shown to provide benefits, as evidenced by their uptake [1,2], we seek to more clearly analyse their current added value to the multiscale application development process, and find previously under-prioritized areas in which software could provide further support.

In this article, we define MCS as software that provides added value during one or more stages of the multiscale application development process, and has an explicitly formulated orientation towards multiscale, multiphysics, multimodel or other coupled applications. Using this definition, we then analyse a representative set of existing MCS in order to establish the current state of the art in MCS, identify the main obstacles preventing a widespread adoption of MCS in science and engineering, and chart a path forward for development of the next-generation MCS. To that end, we start by summarizing the recent developments in MCS in §2, review the common steps in the process of developing a multiscale application in §3, and reflect on the scope, advantages and drawbacks of adopting generic MCS in §4. In §5, we present our analysis approach, followed by an overview of key results from our analysis in §6, and a discussion with conclusion in §7.

## 2. Recent developments in multiscale computing software

Following the formulation of mathematical foundations of multiscale modelling (cf. [3,4] for an overview), computational aspects of multiscale modelling have only recently become the focus of the scientific community. This interest has yielded a number of MCS aiming to ease creation of multiscale models, especially those relying on modern high-performance computing architectures. In particular, emerging exascale computing architectures present both a challenge and an opportunity for MCS development [5]. On the one hand, exascale computers promise to provide an unprecedented compute capacity, most probably required for multiscale modelling. On the other hand, in order to fully harness this capacity, significant algorithmic advances are necessary to handle fault tolerance and robustness, heterogeneity of processors and memory and energy-efficiency, to name a few.

Multiscale modelling is a divide-and-conquer endeavour. Relevant scales, both temporal and spatial, are identified and models developed at each individual scale. These at-scale models are then combined to form a multiscale model. The description of a multiscale model can be formally handled by means of the scale-separation map which defines the individual scales in a multiscale model along with the interactions between scales [6]. The scale separation map is often encoded in the multiscale modelling language (MML), a descriptive language for multiscale model development [7]. More recently, multiscale computing patterns (MCP), higher-level abstractions serving as a basis for more generic MCS software, have been introduced [8]. MCP are categories of multiscale models that exhibit common scale-separation maps and coupling topologies between model components. Example MCP include the Extreme Scaling (ES) pattern where a single at-scale model dominates computational cost within a multiscale model, the Heterogeneous Multiscale Computing (HMC) pattern based on the heterogeneous multiscale method (HMM) [4] where many microscale models are coupled to a macroscale model and launched on-demand, and the Replica Computing (RC) pattern where a large number of individual model ensembles are evaluated under a range of initial conditions.

By their nature, multiscale models are composed of individual at-scale (or single scale) model components. Each at-scale component is frequently a complex parallel software developed over many years. This fact has motivated a shift away from monolithic approaches to multiscale model development and towards more heterogeneous component-based approaches, capable of incorporating existing at-scale models with minimal software modifications. One such approach, the cooperative parallelism programming model, is a task-based multiple-program multiple-data approach to parallel programming [9]. In cooperative parallelism, single unit computation tasks named symponents (a portmanteau of simulation and component) are executed by a runtime system. Symponents are able to interact dynamically with the runtime system to launch, communicate with, and destroy additional symponent calculations. The Co-op MCS implements the cooperative parallelism programming model and leverages the Babel software [10] to integrate symponents together that are written in different programming languages [11,12]. The cooperative parallelism programming model is well-suited for development of multiscale models [13] and the Co-op MCS has been successfully employed for multiscale modelling of materials [14].

Owing to the modularity of the cooperative parallelism approach, developers can easily mix-and-match various at-scale models and incorporate surrogate models to reduce computational cost. For example, adaptive sampling algorithms have been developed within the Co-op system to automatically construct surrogate models during multiscale model evaluation [15]. In adaptive sampling, input and output data obtained from evaluation of at-scale model components are used to construct surrogate models that are stored in a metric-tree database. The surrogate models are much cheaper to compute and can often be evaluated in place of at-scale models with manageable errors. The use of adaptive sampling techniques in a multiscale model can reduce computational cost by several orders of magnitude [14,16]. Moreover, the modular nature of the Co-op system allows for the use of adaptive sampling techniques in any multiscale model developed within the framework. In addition to its implementation in Co-op, the adaptive sampling method has been released in software as the Adaptive Sampling Proxy Application (ASPA) [17].

A modular component-based approach to multiscale modelling is also fundamental to the Multiscale Coupling Library and Environment (MUSCLE) [18]. The MUSCLE software has matured over many years and several different versions have been released. The original MUSCLE is tailored to complex automata modelling and multi-agent computing [19–21]. A subsequent version, MUSCLE 2, is designed for distributed multiscale computation where at-scale model components execute across disparate and potentially geographically separate computers [22]. MUSCLE 2 is able to incorporate at-scale model components written in a variety of programming languages including Java, C, C++, Python and Fortran and is able to directly generate runtime configurations using the MML specification of a multiscale model. Among other things it has been embedded in the VPH Hypermodelling Framework [23]. The newest version, MUSCLE 3, aims to more tightly integrate an extended version of the MML, with better support for dynamic submodel instantiation, surrogate modelling and uncertainty quantification and sensitivity analysis.

Another computational framework for scale-bridging in multiscale modelling is the Hierarchical Multiscale Simulation (HMS) framework [24]. The HMS framework closely follows the HMM for multiscale model construction. HMS combines hierarchies of at-scale model components together and implements a runtime system to schedule and execute at-scale models on available computational resources. Each at-scale model component is taken to be a standalone executable written in any programming language to ease incorporation of existing complex at-scale models into a multiscale model. The HMS framework has been extended to allow for execution of at-scale model components across multiple high-performance computers [25]. In addition, an adaptive sampling algorithm has been introduced into the framework to reduce computational expense [16].

MCS has also arisen within a number of scientific areas, including astrophysics, climate modelling, materials modelling, plasma physics and systems biology [2]. An exhaustive bibliography of multiphysics and multiscale software frameworks through 2015 has been provided in [1]. These MCS are frequently tailored to a particular phenomenon under consideration by each community. Yet, they are often sufficiently generic to be adapted to other areas with minimal effort. One example in astrophysics, the Astrophysical Multipurpose Software Environment (AMUSE), is a Python-based software framework to combine simulation codes together for astrophysical simulations [26]. AMUSE includes a large number of community astrophysics simulation codes to handle gravitational dynamics, stellar evolution, hydrodynamics, and radiative transfer and implements user-friendly features including a unit algebra model to simplify unit-conversions between models in the framework. The AMUSE approach has been proven successful and it now serves as the basis for the Oceanographic Multipurpose Software Environment (OMUSE) for ocean modelling [27]. Other MCS for atmosphere and ocean modelling includes The Earth System Modelling Framework (ESMF), a component-based software platform under development since the early 2000s [28].

For materials science applications, the Exascale Co-design Center for Materials in Extreme Environments (ExMatEx) has developed a number of MCS [29]. ExMatEx has placed a particular emphasis on new exascale computing architectures as an enabler for new approaches to multiscale modelling including task-based computation and adaptive fault-tolerant algorithms [30]. In order to facilitate the creation of new multiscale computing algorithms, ExMatEx has developed a number of proxy apps: simplified at-scale models which mimic the computational workload of more complex models. The proxy apps are designed to be simpler to work with than more complex models for the development of new multiscale computing algorithms. Through the use of the proxy apps, ExMatEx has created and released several software packages for multiscale computing. The Task-based Scale-bridging Code (TaBaSCo) uses Charm++ to execute an adaptive and asynchronous task-based computation of an embedded viscoplasticity model (CoEVP) for the constitutive response of a continuum model of Lagrangian hydrodynamics [31,32]. The software is written to evaluate multiscale computing approaches on the Trinity Advanced Technology System supercomputer, a pre-exascale system at Los Alamos National Laboratory. Another software, the Distributed Database Kriging for Adaptive Sampling (D^2^KAS) implements a redis in-memory data store in combination with locally aware hashing to construct and evaluate kriging surrogate models on-the-fly from at-scale model data [33]. A twist on the adaptive sampling approach is a method which avoids use of a data store entirely, but samples an at-scale model at a set of spatial points at each timestep to construct a surrogate model using Akima splines [34].

In systems biology, the ENteric Immunity Simulator Multi-scale Modelling (ENISI MSM) is a Java-based system for multiscale modelling of immunological processes [35]. ENISI MSM combines together agent-based models, ordinary differential equation-based models, and partial differential equation-based models along with a visualization interface to control the simulation. A recent version of ENISI MSM has been released for high-performance computing environments.

In addition to the task-based integrative frameworks for multiscale modelling described above, there exist a number of coupling frameworks for multiphysics and multiscale modelling. Coupling frameworks are mainly designed to facilitate the exchange of data between different models. Such data exchange occurs at an interface or handshake region between models, as is often the case in partitioned-domain multiscale methods [36]. Coupling frameworks typically implement methods for the interpolation of data between different meshes and parallel data exchange between individual processes in each model. Software implementing this type of coupling includes the Model Coupling Toolkit (MCT) [37] and Multiscale Universal Interface [38]. MCT has been employed in OASIS-MCT to couple two-dimensional fields for climate system modelling [39]. The Macro-Micro-Coupling Tool (MaMiCo) enables coupling molecular dynamics and computational fluid dynamics codes and includes capabilities to perform ensemble sampling of molecular dynamics trajectories to obtain statistically converged flow field quantities [40,41].

Since multiscale models are inherently complex software with individual components which are themselves large-scale parallel applications, tools to aid developers and users of multiscale models are required for multiscale modelling approaches to become widely adopted. For example, the deployment of a multiscale model across multiple high-performance computers where each system has a different compiler suite, MPI version, node configuration, etc., still presents a formidable challenge. Fortunately, these needs are not unique to multiscale modelling, and it is likely that software tools developed in other areas, for example in cloud-based distributed systems, can be easily adopted to form a complete MCS stack.

One effort to address the relative lack of supportive tools for deployment of multiscale models is FabSim [42]. FabSim aims for reproducible execution of complex workflows across multiple high-performance computers and has been successfully applied to multiscale models requiring ensembles of molecular dynamics simulations [43], as well as blood flow [44]. A larger framework like Automated Interactive Infrastructure and Database for computational science (AiiDA) [45] can also be used for this purpose, while tools such as Longbow [46] are suitable alternatives for running single jobs remotely using quick one-liner commands. Workflow packages which can be used to help execute multiscale applications include the Kepler Project [47], the Swift scripting language [48], the Ensemble Toolkit [49] and Parsl [50] which allow for development and execution of parallel workflows involving many individual programmes across clouds and supercomputers.

## 3. Multiscale computing applications: the development process

Before assessing in what ways MCS can benefit the developer of multiscale computing applications, we review a number of common steps we recognize in the development process for multiscale applications.

**Figure 1.**
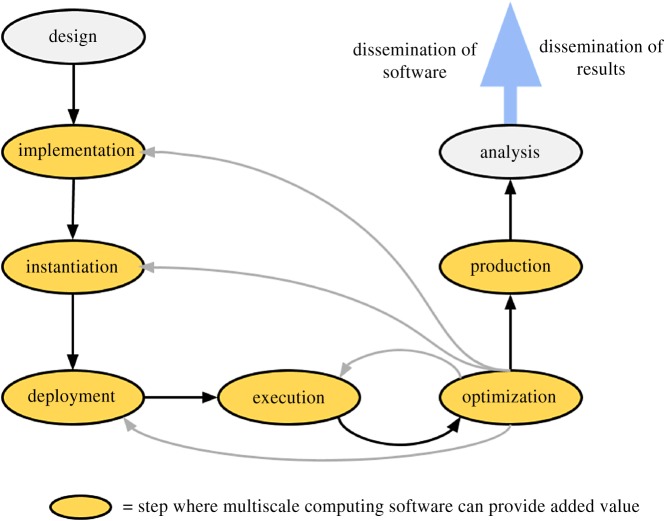
Overview of a typical process for developing multiscale computing applications. (Online version in colour.)

We identify the typical steps required when developing a multiscale computing application in figure 1. Development starts with the design of the conceptual models to address a scientific challenge of interest (Design Step). This includes selecting necessary single-scale models and determining which of these need to be coupled directly. Next, the computational models are adapted, and coupling mechanisms are implemented to facilitate the transport of data between the submodels (Implementation Step). This can, for example, be done using coupling libraries or workflow tools. Once the single-scale models and coupling mechanisms have been established, the implementation can be applied to the specific scientific problem of interest (Instantiation). This includes adding relevant data and parameters, e.g. force field definitions and initial particle configurations for a multiscale molecular application.

After Instantiation, the application is made operational at the target platform (e.g. cluster, cloud or supercomputer, Deployment Step), upon which it is (initially) run (Execution Step). Deployment is complicated due to the fact that various single-scale models and their coupling have to be orchestrated, potentially on a heterogeneous platform (CPU/GPU supercomputer) or even multiple platforms (combining clusters or working in a cloud). After the initial run, the application is usually subject to a cycle of further optimizations (Optimization Step) and executions (or repetitions of earlier steps as needed). Optimization in the context of this paper refers to bolstering the scientific and technical quality of the application such that it becomes suitable for use in production runs. This may involve fixing verification or validation issues that arose during execution, rerunning the application multiple times to test the sensitivity of key parameters, or to check the propagation of uncertainties in the model. Once the application has been sufficiently optimized, researchers proceed with performing the main runs (Production Step) and analyse its output data (Analysis Step). Lastly, researchers disseminate their work by publishing key results, and/or the software approach that they have developed to obtain these results (Dissemination Step).

## 4. The role of multiscale computing software

We define MCS as software which adds value during one or more stages of the multiscale application development process, and has an explicitly formulated orientation towards multiscale, multiphysics, multimodel or coupled applications. Arguably, there are six steps in the multiscale application development process where MCS can provide added value: (1) Implementation, (2) Instantiation, (3) Deployment, (4) Execution, (5) Optimization and (6) Production.

In this work, we investigate the added value of a range of MCS, attempting to include an example of each type that is commonly used. Because the number of MCS packages is very large, our analysis is not exhaustive, but focuses on major examples of each specific type that we are aware of, and that are publicly available. For example, our analysis of the potential added value of the OpenFOAM multiphysics code [51] will apply to a large extent to other multiphysics codes, such as Elmer or LAMMPS [52]. Likewise, analysis concerning the widely used OASIS-MCT coupler similarly helps to determine the added value of other couplers, such as C-Coupler1 [53] or YAC [54].

### 

#### (i) Scope

An important aspect of MCS is the intended scope of the software. Here we briefly reflect on a few relevant scopes of applicability for these tools, from more specific to more generic. Software can be instance-specific (e.g. written ad-hoc for a single run or typed into an interactive terminal), problem-specific (e.g. custom-made for a clay-polymer MD simulation), system-specific (e.g. tailored for MD simulations), discipline-specific (e.g. intended for materials science applications) or generic. More generic software tends to have a stronger focus on ease of reuse, serves a larger community and tends to get scrutiny from people with a wider range of academic backgrounds. However, a major drawback is the need to engineer the software for a wider range of possible use cases, which may increase the effort required to develop more generic MCS.

Reusability may be limited not only to the extent that MCS is generic from a scientific perspective. Other limitations, such as restrictions on supported codes, programming languages, user types, operating systems or resource platforms can further limit both the reusability of MCS, and other aspects such as the maximum attainable size of its user community.

#### (ii) Advantages

Many different kinds of added value can be provided by MCS, but for the purposes of this work we place any added value advertised by these tools within four categories, each of which may apply to the aforementioned six steps in the development process.

Software may help *Curate* multiscale applications, e.g. by making activities more reproducible, more organized, more transparent and/or easier to scrutinize. Software may help to *Accelerate* multiscale applications by speeding up the progress in a process step, or to *Simplify* by reducing the amount of skills and knowledge needed to perform that step. Lastly, MCS may *Expand* the range of possibilities for the developer by introducing alternative approaches, or by providing more flexible use of existing ones.

#### (iii) Drawbacks

Adopting generic software for multiscale computing provides clear benefits, but choosing more generic tools over more specific ones comes with a range of drawbacks as well. These drawbacks are described in detail in table 1.

**Table 1.** Overview of drawbacks when using generic MCS

type	description of drawback	example means of mitigation
adoption overhead	it takes time to understand and set up software which is written by others	good and simple build systems, clear documentation, tutorials
application overhead	it takes time to introduce domain- or problem-specific in a generic setting, and to modify generic code to facilitate an unexpected situation	make MCS non-intrusive, limit the range of features provided by the MCS, clear documentation, tutorials
search overhead	it can take time to find the right software (or it might not exist at all)	create search directories, pursue good citation practices of directly used and closely related MCS in scientific articles
increased support requirements	more support effort is necessary due to a larger community size, leading to less support per user	establish a self-supporting user community
lack of control and/or ownership	development is frequently managed by others, reduced academic credit due to not developing own tools, no control over the software installation in the case of software as a service	make code open-source, support branching developments and spin-offs, avoid centralized installations, make a clear case against reinventing the wheel

We argue that the first four of these five drawbacks apply less frequently when choosing domain- or system-specific MCS, while the fifth drawback applies to any type of externally owned or controlled software. Though a detailed drawback analysis is beyond the scope of this work, we do recommend that application developers consider these possible drawbacks prior to adopting new MCS, and that MCS developers attempt to identify and mitigate the most serious drawbacks in their software.

## 5. Analysis approach

We have collectively gathered data on a range of MCS, allowing all authors to submit information about specific tools into a database using a Google Form. Each tool was examined by at least two of the authors. An empty example form is provided in the electronic supplementary information as a reference. As a starting point, we investigated a subset of the software presented by Groen *et al*. [2], upon which we then manually searched for more recent MCS. We recorded 26 tools in total, and chose to analyse 21 of them. Of the analysed tools, 20 of them are freely available to the public, while 1 tool (HMS) was freely available to the authors, and is expected to be released freely to the public in early 2019. The other five tools were omitted either because we could not access their public website or because they had been superseded with newer tools.

## 6. Results

### (a) High-level overview

We provide a brief overview of the scope and supported platforms and patterns in table 2. Here, we find that C++ is the most widely supported language, although Python is also quite prevalent. In terms of MCPs, we see a clear segregation of tools, with a large number of tools providing support for one specific MCP. This is interesting, because the MCPs were introduced well after many of these tools were established [8].

**Table 2.** Summary of MCS scope and platforms. The scope is given in the second column, supported programming languages in the third column, and supported multiscale computing patterns (Extreme Scaling (ES), Heterogeneous Multiscale Computing (HMC) or Replica Computing (RC)) in the fourth column.

name	scope	supported languages	supported patterns
ASPA	generic	C++	HMC
Amuse	discipline-specific	Python	ES,HMC,RC
Cactus	generic	Custom (language of choice)	HMC
CoHMM/D2KAS	generic	C++	HMC
CouPE	generic	C++, FORTRAN, Wrappers for C/C++/Fortran modules exist (explains my answer here)	ES,HMC
ELMER	system-specific	C++, FORTRAN, C	ES
ENISI MSM	discipline-specific	Java	ES
ESMF	discipline-specific	C++, FORTRAN	ES,HMC,RC
FLASH	discipline-specific	FORTRAN	ES
FabSim	generic	Python	ES,HMC,RC
HMS	generic	Python, C++, FORTRAN	RC
MOOSE	system-specific	C++	ES,HMC
MPWide	generic	Python, C++, C	ES
MUI	generic	C++	ES
MUSCLE2	generic	Python, C++, Java, FORTRAN, Ruby, C, Matlab	ES
MaMiCo	system-specific	C++, command-line (e.g. bash), SCons for compiling	ES,HMC
OASIS3-MCT_3.0	discipline-specific	FORTRAN, C	ES
OMFIT	discipline-specific	Python	ES,HMC,RC
Parsl	generic	Python	RC
Swift	generic	Domain-specific or bespoke language	ES,HMC,RC
TabaSCo	discipline-specific	C++, CHARM++	HMC

**Figure 2.**
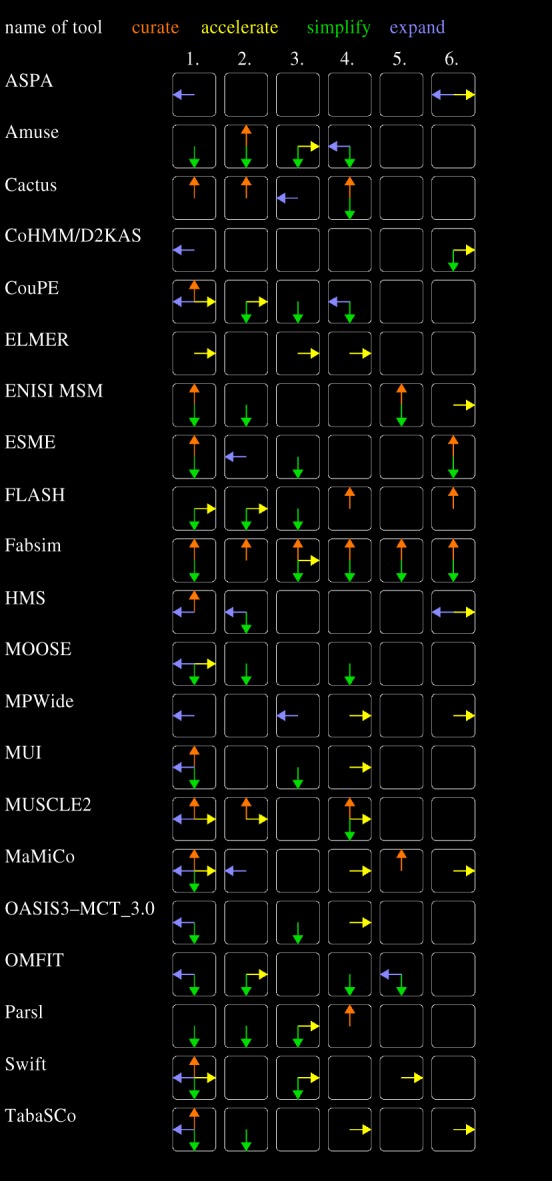
Overview of added value of the software tools. Here, the tools are provided one per row and the number of each relevant development step in each column (respectively (1) Implementation, (2) Instantiation, (3) Deployment, (4) Execution, (5) Optimization and (6) Production). Tools are sorted alphabetically. (Online version in colour.)

We provide an overview of the added values from the tools in figure 2, using the approach we introduced earlier. This list includes 11 generic toolkits, 7 discipline-specific toolkits and 3 system-specific toolkits. In this figure, we can quickly distinguish several things. Firstly, tools that serve more development steps are shown with more filled boxes in the figure. Entries that contain all (or nearly all) filled boxes provide support throughout the development process, while entries with fewer filled boxes are more specific in their purpose. Using more specific tools can mitigate the adoption overhead, as there are fewer development steps that need to be incorporated. Second, the number of arrows inside each box helps indicate the completeness of added values a tool provides in that step. For example, MUSCLE 2 and MPWide both provide added value in the implementation step, but whereas MPWide only expands the range of options in this step, MUSCLE 2 also delivers curation and acceleration benefits due to it providing a more structured framework. This does not necessarily mean that MUSCLE 2 is the better option in all cases; the choice between the two may partially depend on the application need for curation and acceleration in the implementation step.

We provide summary statistics for the added values in table 3. Although our review is far from exhaustive, and many tools we examine have counterparts that are somewhat similar (e.g. OpenFOAM to Elmer, AiiDA to FabSim), it does give a general impression of which areas of added value are targeted to which extent. Based on the results, we find that the tools in our study particularly focus on the Implementation step, and less on the Instantiation and Execution steps. In terms of added values, we recognize a strong focus on simplification and curation, with many of the more recently emerged tools particularly targeting the latter.

**Table 3.** Summation of added values from MCS in each development step

step	no. curate	no. accelerate	no. simplify	no. expand
implementation	10	5	14	12
instantiation	5	2	9	2
deployment	2	6	8	1
execution	4	8	8	2
optimization	4	1	6	1
production	2	7	1	2
total	27	29	46	20

The table also exposes a range of clear added value gaps in our examined tools. We did not record any added value towards accelerating the optimization step (and relatively little added value overall), and tools provide even fewer features that help users during the production step. The strong focus of tools on earlier phases of the development process, and relative lack of focus on later phases, could be seen as an indicator that multiscale computing as a discipline has not yet fully matured.

### (b) Detailed analysis of selected tools

#### (i) Adaptive Sampling Proxy Application

The Adaptive Sampling Proxy Application (ASPA) is a toolkit for automated construction of surrogate models within a multiscale model hierarchy. It allows developers across disciplines to construct kriging surrogate models on-the-fly using data obtained from the evaluation of at-scale model components of a multiscale model. ASPA uses a local kriging strategy to limit the amount of data incorporated in an individual surrogate model and contains a metric tree database to store the collection of surrogate models and allow for their quick retrieval. The adaptive sampling method is intended for use in applications that fit the HMC pattern, specifically for cases where a surrogate model is able to approximate the microscale model well for particular model inputs.

*Implementation*. Expand. Expands the concept of multiscale simulation software. In addition to software consisting solely of coupled at-scale models, ASPA introduces a database to store surrogate models constructed using model output data and allows the surrogate models to be incrementally updated and quickly evaluated, including an error estimate.

*Instantiation, Deployment, Execution and Optimization*. To the best of our knowledge, ASPA does not directly add value on these steps of the development process.

*Production*. Accelerate and Expand. Accelerates evaluation of computationally demanding multiscale models and enables using surrogates in production.

#### (ii) MUSCLE 2

The MUltiScale Coupling Library and Environment 2 establishes couplings between at-scale model components in a systematic and discipline-agnostic manner. It takes a description of the model in terms of components and conduits between them, and executes the simulation accordingly by starting processes and opening TCP connections. Components must be linked with the MUSCLE 2 library, available in a range of languages, to be usable. Although it is not impossible to dynamically instantiate model components separately, MUSCLE 2 provides no support for this, and is mostly geared towards ES applications.

*Implementation*. Curate, Accelerate and Expand. Requires the model structure to be clearly described, takes care of network communications and enables coupling of very diverse models.

*Instantiation*. Curate and Accelerate. Unifies multiscale application definition and parameter values in a single archivable file.

*Execution* Curate, Accelerate, Simplify. Model description includes directions for starting the full application. Can start up all components locally in a single command, automatically establishes network connections, and logs what was done.

*Deployment, Optimization and Production*. To the best of our knowledge, MUSCLE 2 does not directly add value on these steps of the development process.

#### (iii) OASIS3-MCT

OASIS3-MCT is a so-called *coupler* which enables the coupling of models with a focus on climate model components. It originates from the Centre Européen de Recherche et Formation Avancée en Calcul Scientifique (CERFACS). OASIS3-MCT provides a high level of parallelism, and is particularly optimized for efficient interpolation and regridding as well as data exchange in coupled mesh-based applications.

*Implementation*. Expand. OASIS3-MCT supports one-to-many concurrent couplings, a feature which is relatively rare in other toolkits.

*Deployment*. Simplify. Provides a wrapper which makes deployment of all models easier.

*Execution*. Accelerate. OASIS3-MCT supports parallel coupling channels using MPI, clearly improving performance compared to single MPI channels, or TCP/file I/O communications. The toolkit as a whole is also heavily optimized to be fast.

*Instantiation, Optimization and Production*. To the best of our knowledge, OASIS3-MCT does not directly add value on these steps of the development process.

#### (iv) OMFIT

OMFIT is a model coupling framework which features a GUI, and is used extensively in the Fusion community. Its implementation is generic, and supports the use of parallel codes on HPC resources. It has a large range of supported modules built-in, which provide both physics solvers as well as other functionalities such a integrations with data sources and visualization tools.

*Implementation*. Simplify, Expand. Provides a wide range of modules that can be easily coupled, and a GUI to simplify the process of making couplings.

*Instantiation*. Accelerate, Simplify. Integrates with a range of experimental databases, which makes instantiation simpler and faster in a range of cases.

*Execution*. Simplify. OMFIT allows predefining coupling schemes, and simplify doing test runs in that way.

*Optimization*. Simplify, Expand. Supports a range of analysis and visualization techniques to make this step more flexible and simpler.

*Deployment and Production*. To the best of our knowledge, OMFIT does not directly add value on these steps of the development process.

#### (v) Parsl

Parsl is a Python-based parallel scripting library that supports development and execution of asynchronous and parallel data-oriented workflows (dataflows). These workflows glue together existing executables (called Apps) and Python functions with control logic written in Python. Parsl brings implicit parallel execution to standard Python scripts.

*Implementation*. Curate, Simplify, Expand. Provides a dependency-driven workflow model. Allows creation of complex workflow using any infrastructure (from laptop to supercomputer) through one script. Enables the creation of interactive data-intensive workflows.

*Instantiation*. Simplify. Simplifies the passage of data between models.

*Deployment*. Accelerate, Simplify. Single scripts map directly to a range of resource platforms.

*Execution*. Curate. Provides a range of sophisticated data handling and error management features.

*Optimization and Production*. To the best of our knowledge, Parsl does not directly add value on these steps of the development process.

#### (vi) Macro-Micro-Coupling

The Macro-Micro-Coupling Tool (MaMiCo) [40,41] attempts to ease the development of and share existing coupling algorithms for particle-mesh, in particular for molecular-continuum, flow simulations and is therefore a system-specific MCS. Separating continuum and molecular dynamics (MD) solvers from the actual coupling algorithm via strict interfacing and also separating coupling steps in a modular way within MaMiCo, arbitrary solvers can be plugged together. The software supports execution on distributed-memory platforms using MPI, is written in C++ and uses SCons for compiling.

*Implementation*. Curate, Accelerate, Simplify and Expand. MaMiCo features well-defined interfaces to support among others debugging and unit/integration testing. After a certain accustomization phase, this also accelerates and simplifies code development. Accordingly, new algorithms to couple MD and continuum solvers can be easily incorporated as demonstrated in [40] (expansion).

*Instantiation*. Expand. Once a new coupling algorithm for a particular flow problem has been incorporated, this coupling can be evaluated using any interfaced particle/continuum package immediately.

*Deployment*. None.

*Execution*. Accelerate. Through the multi-instance sampling in MaMiCo [40], faster time-to-solution is reached, although at higher compute cost. Incorporation of noise filters is a work in progress and is meant to further accelerate sampling/noise reduction in MD.

*Optimization*. Curate. Standardized coarse-grained output is provided through MaMiCo (csv and vtk formats), allowing to compare results for different couplings.

*Production*. See acceleration aspect for multiscale software execution above.

#### (vii) FabSim

FabSim is an automation environment which is optimized for curating complex multiscale workflows and providing one-liner access to perform simulations on remote machines. Its base implementation is generic, and has been used in disciplines ranging from materials to blood flow and migration. It is designed to be easy to modify, and has resulted in several domain-specific spin-off tools (e.g. FabMD [43] and FabFlee [55]) over the years.

*Implementation*. Curate and Simplify. Easily combine execution patterns. Curate workflow building blocks.

*Instantiation*. Curate. Curate collections of simulation input.

*Deployment*. Curate and Accelerate. Automate deployment. Reuse working configs from other users.

*Execution*. Curate and Simplify. Curate simulation output and environment. Simplify execution on remote resources.

*Optimization*. Curate and Simplify. Curate output and environment. One-liner commands for parameter explorations.

*Production*. Curate. Curate output and environment. Curate multi-machine workflows in single commands.

## 7. Conclusion

Several conclusions can be drawn from our analysis. First, the availability of MCS has become considerably broader since 2014 [2], with many of the newer tools aiming explicitly to simplify application development. Second, Python has now become one of the leading platforms to help facilitate multiscale applications (e.g. see FabSim, OMFIT and Parsl for recent examples). Third, in terms of generality, we now find generic MCS being applied in all major computational research disciplines. However, the tools remain quite specific in other aspects: most MCS are far from language-agnostic and 16 of the 21 tools are intended for a subset of applications that fit one or two particular MCPs [8].

Until now, the majority of the MCS development has focused on integrating frameworks to combine at-scale models together to form a multiscale model. As the multiscale computing field remains still quite immature, there has been a corresponding lack of development of tools to ease deployment, configuration, debugging, profiling, optimization and visualization of multiscale models. As a consequence, we find in our analysis that relatively few tools provide added value in the later steps in the development process (especially deployment, optimization and production). These steps are both labour-intensive and crucial for the long-term success of multiscale applications, and the introduction of mature MCS there may drive the research impact in the field as a whole.

Within our work we also briefly reflect on the (frequently under-documented) drawbacks associated with MCS. A systematic analysis of drawbacks, describing the trade-offs expected when adopting the software, does not solely serve the community as a whole. It can also give a much clearer justification to the existence of individual tools, particularly when two tools with similar added values provide these benefits with substantially different kinds of drawbacks.

Major progress has been made towards providing discipline-agnostic MCS. Now, our next targets should be to address the previously overlooked parts of application development, and to more clearly present and curate the adoption drawbacks and benefits to the users.

## Supplementary Material

Survey form

## Supplementary Material

Raw survey data

## Supplementary Material

Review data

## Data accessibility

This article has no additional data.
