# Supplementary material for: Mastering the scales: a survey on the benefits of multiscale computing software
Source: Philos Trans A Math Phys Eng Sci. 2019 Feb 18;377(2142):20180147. doi: 10.1098/rsta.2018.0147 (PMC6388006; doi:10.1098/rsta.2018.0147)
Supplement: Survey form [file rsta20180147supp1.pdf]

Review of Multiscale Computing Software

This form serves to gather input regarding the added value of specific software toolkits for multiscale computing.

Please fill this form in ONCE for every multiscale computing toolkit.

In this first section, please enter a range of essential information in regards to the toolkit.

1. **Who is submitting this entry**
Mark only one oval.

- ( ) Derek
- ( ) Jaroslaw
- ( ) Philipp
- ( ) Lourens
- ( ) Kenneth
- ( ) Diana
- ( ) Other: ______________________

2. **Name of the software tool**

____

3. **Main website of the software tool (or repository)**

____

4. **DOI of paper related to software**

____

5. **Intended scope of the software**
Check all that apply.

- [ ] Generic
- [ ] Discipline-specific (e.g., only for astrophysics, biomedicine)
- [ ] System-specific (e.g., only for particle-continuum coupling, or only for molecular simulations)
- [ ] Code-specific (e.g., only to be used with LAMMPS or GADGET)
- [ ] Platform-specific (only tick if it is intended NOT for a generic Linux-based platform)

6. **Language of the software (application developer side)**
Check all that apply.

- [ ] Domain-specific or bespoke language
- [ ] Python
- [ ] C++
- [ ] Graphical User Interface
- [ ] Java
- [ ] FORTRAN
- [ ] command-line (e.g., bash)
- [ ] Other: ______________________

7. **The language the software itself is written in is:**
Check all that apply.

- [ ] Python
- [ ] C++
- [ ] C
- [ ] Java
- [ ] FORTRAN
- [ ] Perl
- [ ] Domain-specific or bespoke language
- [ ] Other: ______________________

8. **For which multiscale computing pattern(s) is the software applicable**
Check all that apply.

- [ ] Extreme Scaling
- [ ] Heterogeneous Multiscale Computing (or Hierarchical Multiscale Modelling)
- [ ] Replica Computing
- [ ] Other: ______________________

Characterizing added value

On this page we attempt to characterize the added value of your chosen software tool for multiscale computing, at various steps of the multiscale application development process.

To make this clear, we define a set procedure of steps for multiscale application development (see first image), and a set categorization of added value types that can be provided by multiscale computing software (see second image).

Steps in developing multiscale computing software

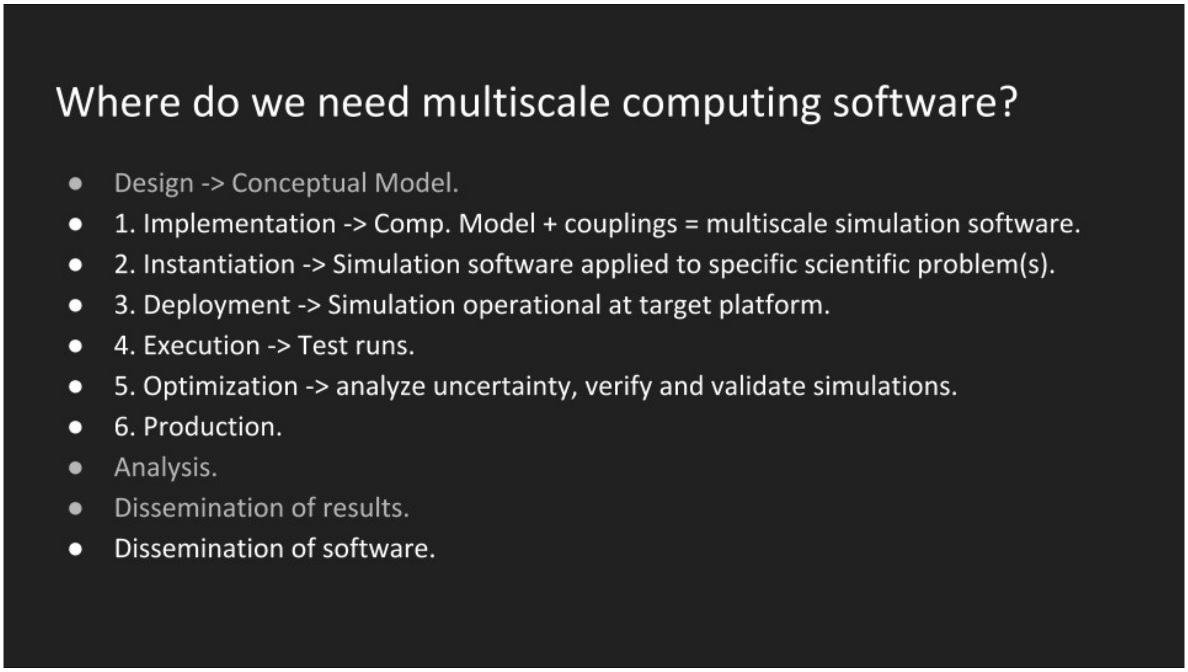

The four types of added value.

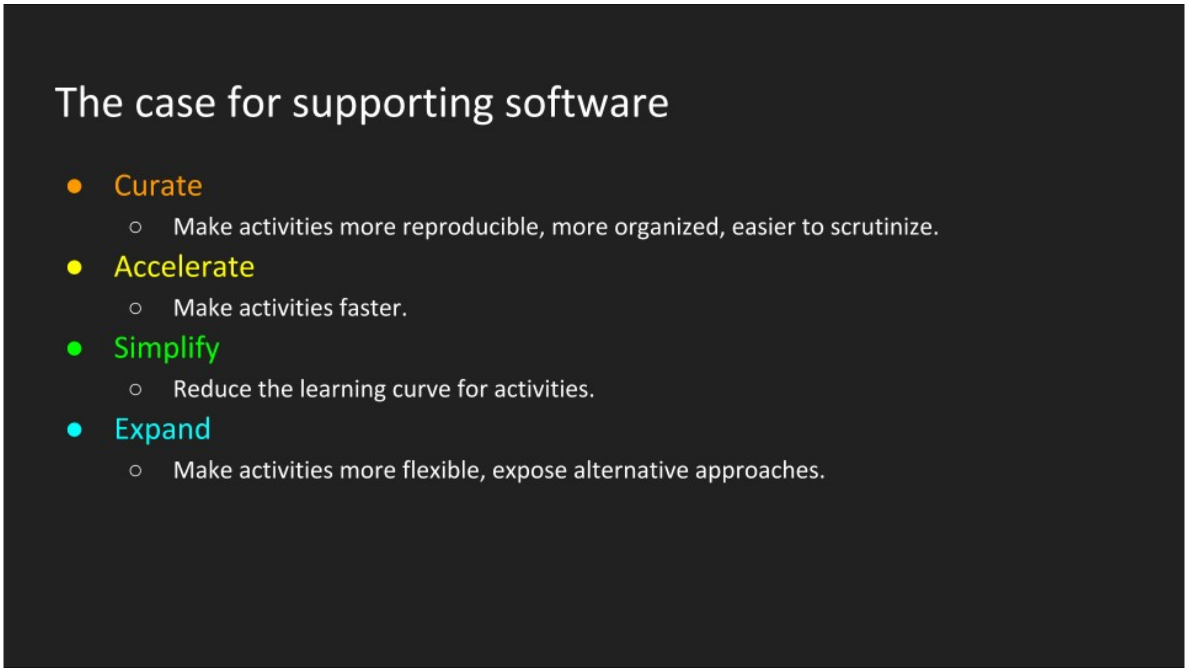

9. **1. Implementation -> Comp. Model + couplings = multiscale simulation software.**

Check all that apply.

- [ ] Curate
- [ ] Accelerate
- [ ] Simplify
- [ ] Expand

10. **Comments on Implementation (optional)**

11. **2. Instantiation -> Simulation software applied to specific scientific problem(s).**
Check all that apply.

- [ ] Curate
- [ ] Accelerate
- [ ] Simplify
- [ ] Expand

12. **Comments on Instantiation (optional)**

13. **3. Deployment -> Simulation operational at target platform.**
Check all that apply.

- [ ] Curate
- [ ] Accelerate
- [ ] Simplify
- [ ] Expand

14. **Comments on Deployment (optional)**

15. **4. Execution -> Test runs.**
Check all that apply.

- [ ] Curate
- [ ] Accelerate
- [ ] Simplify
- [ ] Expand

16. **Comments on Execution/Test runs (optional)**

17. **5. Optimization -> analyze uncertainty, verify and validate simulations.**
Check all that apply.

- [ ] Curate
- [ ] Accelerate
- [ ] Simplify
- [ ] Expand

18. **Comments on Optimization (optional)**

19. **6. Production.**
Check all that apply.

- [ ] Curate
- [ ] Accelerate
- [ ] Simplify
- [ ] Expand

20. **Comments on Production (optional)**

Non-essential additional information

This last section serves to store any additional details that you would like to provide.

21. **Short description of the toolkit**

22. **Any other details you wish to share in regards to the toolkit**

Powered by
Google Forms
